# Application of Diffusion Tensor Imaging (DTI) in the Diagnosis of HIV-Associated Neurocognitive Disorder (HAND): A Meta-Analysis and a System Review

**DOI:** 10.3389/fneur.2022.898191

**Published:** 2022-07-07

**Authors:** Juming Ma, Xue Yang, Fan Xu, Hongjun Li

**Affiliations:** ^1^Department of Radiology, Beijing YouAn Hospital, Capital Medical University, Beijing, China; ^2^Beijing Advanced Innovation Centre for Biomedical Engineering, Beihang University, Beijing, China

**Keywords:** meta-analysis, HIV-associated neurocognitive disorder (HAND), diffusion tensor imaging (DTI), fractional anisotropy (FA), mean diffusivity (MD)

## Abstract

**Background:**

The patients with HIV-associated neurocognitive disorder (HAND) are often accompanied by white matter structure damage. Diffusion tensor imaging (DTI) is an important tool to detect white matter structural damage. However, the changes in DTI values reported in many studies are diverse in different white matter fiber tracts and brain regions.

**Purpose:**

Our research is dedicated to evaluating the consistency and difference of the correlation between HAND and DTI measures in different studies. Additionally, the value of DTI in HAND evaluation is used to obtain consensus and independent conclusions between studies.

**Methods:**

We searched PubMed and Web of Science to collect relevant studies using DTI for the diagnosis of HAND. After screening and evaluating the search results, meta-analysis is used for quantitative research on data. Articles that cannot collect data but meet the research relevance will be subjected to a system review.

**Results:**

The meta-analysis shows that the HAND group has lower fractional anisotropy (standardized mean difference = −0.57 *p* < 0.0001) and higher mean diffusivity (standardized mean difference = 0.04 *p* < 0.0001) than the healthy control group in corpus callosum. In other white matter fibers, we found similar changes in fractional anisotropy (standardized mean difference = −1.18 *p* < 0.0001) and mean diffusivity (standardized mean difference = 0.69 *p* < 0.0001). However, the heterogeneity (represented by I^2^) between the studies is high (in corpus callosum 94, 88%, in other matter fibers 95, 81%). After subgroup analysis, the heterogeneity is obtained as 19.5, 40.7% (FA, MD in corpus callosum) and 0, 0% (FA, MD among other white matter fibers).

**Conclusion:**

The changes in white matter fibers in patients with HAND are statistically significant at the observation level of DTI compared with healthy people. The differences between the studies are mainly derived from demographics, start and maintenance time of antiretroviral therapy, differences in nadir CD4+T cells, and the use of different neurocognitive function scales. As an effective method to detect the changes in white matter fibers, DTI is of great significance for the diagnosis of HAND, but there are still some shortcomings. In the absence of neurocognitive function scales, independent diagnosis remains difficult.

**Systematic Review Registration:** https://inplasy.com/inplasy-2021-10-0079/.

## Background

The emergence of combined antiretroviral therapy (cART) has resulted in a significant increase in the life expectancy of patients with acquired immunodeficiency syndrome (AIDS) in the past two decades (1). The increase in the average age of HIV seropositive people has also led to age-related complications. The incidence of disease is increasing, and HAND is one of the most serious complications (2). Increased life expectancy in people living with HIV is clearly a major factor in their increased incidence of neurodegenerative diseases, but this often masks the important effect of HIV on neuronal damage (3). The Frascati standard revised in 2007 is currently the most widely used diagnostic standard for HAND. The standard divides HAND into three levels according to the severity of cognitive impairment (4): asymptomatic neurocognitive impairment (ANI), mild neurocognitive disorder (MND), and HIV-associated dementia (HAD). To date, the pathogenesis of HAND has not been fully defined. It is thought to be caused by a combination of factors such as chronic immune activation, indirect effects of inflammatory responses, direct neurotoxic effects of viral proteins, and vascular damage (5, 6). Despite the cART treatment, the microglia and astrocytes of the central nervous system (CNS) can still serve as HIV reservoirs, which in turn can cause continuous damage to neurons (7). Therefore, cART can delay the progression of HAD, but cannot completely prevent the occurrence of HAND, and some studies (8, 9) have shown that the severity of cognitive impairment is not related to the cerebrospinal fluid and peripheral viral load, but is related to the lowest value of CD4+T cells. In addition, the current mechanisms related to HAND include the followings: the toxic effects of ART, changes in the permeability of the blood–brain barrier, abnormal lipid metabolism, and oxidative stress. The various mechanisms are closely linked and influence each other. Moreover, bad lifestyle habits such as smoking, alcohol abuse, and drug use can not only affect the cognitive function of HIV-infected persons through direct toxic effects on CNS, but also impair cognitive function through mutual pharmacological effects with cART (10).

In the diagnosis of cognitive impairment, both cognitive scales and imaging tests play an important role. The International HIV Dementia Scale (IHDS) is currently widely used, which includes attention, memory recall, and psychomotor speed. Compared with the Frascati standard, IHDS shows better sensitivity and specificity (11).

Diffusion tensor imaging uses the anisotropy of the dispersion of water molecules inside the white matter fibers (WMFs) and uses the average diffusion direction information of adjacent voxels through the continuous tracer fiber allocation algorithm to reconstruct the structure and shape of the WMF, and then, imaging method shows the three-dimensional distribution of white matter fiber bundles *in vivo* (12, 13). Among the parameters of DTI, fractional anisotropy (FA) and mean diffusivity (MD) represent the proportion of water molecule anisotropy in the whole diffusion tensor and the average diffusion capacity of water molecules in different directions, respectively. The axial diffusion coefficient (AD) and radial diffusion coefficient (RD) describe the random motion diffusion amplitude of water molecules and the diffusion direction perpendicular to the axon, respectively. They are also commonly used parameters in various studies (14–16). A majority of studies (17–19) reported that compared with healthy controls, patients with HAND had a statistically significant decrease in FA value and an increase in MD value, suggesting that patients with cognitive impairment may have lesions of WMF. The areas where the FA value decreases are common in the genu of the corpus callosum (GCC), the splenium of corpus callosum (SCC), bilateral superior longitudinal fasciculus (SLF), bilateral inferior fronto-occipital fasciculus (IFOF), corticospinal tract (CST), bilateral uncinate fasciculus (UF), etc. The areas where the MD value decreases are common in bilateral external capsules (EC), bilateral corpus callosum (CC), left fornix (FX), left anterior radiating coronal area, left posterior thalamic radiation (PTR), and left EC (20, 21). Correlations between cognitive impairment and changes in DTI parameters are complex. Some studies (22–25) have shown that the degree of cognitive impairment has a significant statistical correlation with the decrease in FA value and the increase in MD value. The decrease in FA value has the most significant correlation with cognitive impairment. However, another part of the research (26–28) shows that in the patients of the same cognitive level group, the FA value and MD value changes are complicated, and there may even be opposite changes in adjacent areas, accompanied by axial diffusivity AD and RD changes (29, 30). There are studies (31, 32) reported that with the continuation of cART, the FA value will increase and the MD value will decrease, which is related to the recovery of CD4+ T cell levels.

There are also differences in the application technology of DTI between different studies. The more common analysis methods of DTI are the traditional voxel-based analysis (VBA) method and the skeleton-based spatial statistics (tract-based spatial statistics, TBSS) method, and the latter is more widely used. Compared with the traditional voxel-based measurement method, Fixel-based analysis (FBA) (33), which has been applied recently, can reduce the interference of extracellular free water generated by intracranial lesions and can more accurately display the content of intracellular edema. Of course, interference can also be reduced by performing free water corrections (34), all of which are intended to provide more reliable WM integrity measurements.

Therefore, based on the diversity of parameters, demographic application models, and outcomes of DTI in the diagnosis of HAND, we believe that it is meaningful to conduct a meta-analysis and system review of related studies. We will examine the correlations between DTI parameters, diagnostic criteria for cognitive impairment, and serum viral markers to obtain consensus and independent views between studies.

## Materials and Methods

### Article Retrieval

To summarize the relevant research literature on the related application of DTI in HAND, we used an advanced search on the PubMed database and the Web of Science database on 30 September 2021, through use the term of “ (‘HIV-associated neurocognitve disorder' [MeSH Terms] OR ‘HAND' [All Fields] OR ‘HIV' [All Fields]) AND (‘Diffusion Tensor Imaging' [MeSH Terms] OR ‘DTI' [All Fields] OR ‘Magnetic Resonance Imaging' [All Fields] OR ‘neuroimaging' [All Fields]),” (((TS=(DTI) OR TS=(Diffusion Tensor Imaging) OR TS=(MRI) OR TS=(neuroimaging)) AND (TS=(HIV) OR TS=(HIV-associated neurocognitive disorder) OR TS=(HAND) OR TS=(cognitive impairment))). The search criteria are the documents from 2013 to 2021. The literature search produced a total of 104 records, by reading the abstract, of which 67 records were excluded due to the following reasons: (1) duplicate literature, (2) the study was conducted in mice or rhesus monkeys, (3) the records were case reports, and (4) the records were reviews, (5) the study has no control group, (6) the research participants are not infected with HIV virus, and (7) the literature topics and imaging examinations are less related.

### Inclusion and Exclusion Criteria

**Figure 1:**
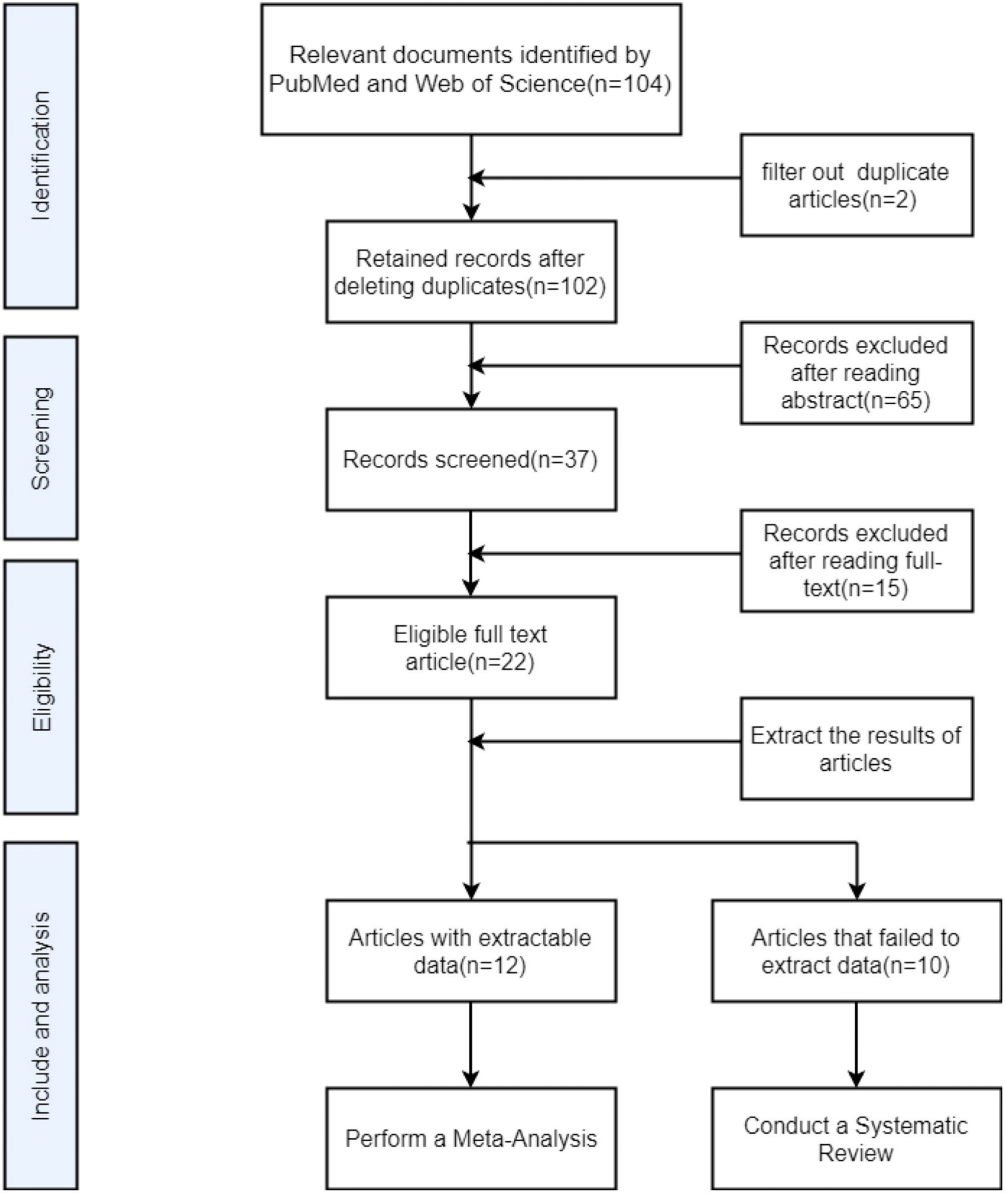
Flow chart for searching and screening articles included in meta-analysis and system review.

We retrieved a total of 37 eligible full-text articles, all of which belonged to cross-sectional studies or cohort studies. We read through the full text, and once again ruled out 15 articles due to the following reasons: 1. Research participants also suffer from neurodegenerative diseases such as Alzheimer's disease, Parkinson's disease, cerebrovascular disease. 2. Research participants also suffer from syphilis, hepatitis B, hepatitis C, and other diseases. 3. multi-modal research but incomplete experimental results involving DTI. 4. Research using resting state functional magnetic resonance imaging or 3D-T1 mapping and other sequences. 5. The main research direction is the aging of brain structure status. Of the remaining 22 articles, 12 articles can be collected with comprehensive and complete FA and MD values, including the CC, FX, anterior commissure (AC), association fibers, and projection fibers. We included these 12 articles in the meta-analysis. Due to the unique role of the CC in brain structure and cognitive function, the FA and MD values were separately extracted in the form of mean ± SDs. All data are from the table of the article. The remaining 10 articles cannot be extracted with complete data due to the following reasons: (1) The researcher only reported the FA value and MD value of the whole brain or a part of the brain area; (2) the researcher's main direction of study is the value of DTI parameters and serum sickness. There will be the correlation between the viral indicators and the test scores of each neurocognitive scale. We conducted a system review of these 10 articles (Figure 1).

### Meta-Analysis and System Review

For the 22 articles included in the meta-analysis and system review, two people independently completed the quality evaluation and data extraction. When the results were disputed, a third person participated in the completion. Since the articles included in the analysis are cross-sectional or cohort studies, the quality evaluation system adopts the Newcastle–Ottawa Scale (NOS), including 7 indicators such as participant selection, exposure, and comparability control for important factor and for quality evaluation. We summarize all relevant research in inclusion analysis, including the statistical characteristics, research type, basic parameters of magnetic resonance, treatment or not, whether neuropsychology (NP) and cognitive function scale test, and region of interest (ROI) (Table 1). Due to the unique role of the CC in brain structure and cognitive functions, we extract the FA and MD values of the CC separately and jointly extract the FA and MD values of the FX, AC, and other WMF. Because there are great differences in the selected regions of interest between different studies, we selected several regions of interest with great commonalities. The number of included samples is less than that of CC, but it is also meaningful. Use Review Manager 5.3 software to detect heterogeneity, sensitivity, and risk of bias on the extracted data (58). Random-effects models are used to obtain standardized average differences (SMDs), which is obtained by dividing the estimated mean difference between the two groups by the mean standard deviation. For the continuous variables used in this study, when SMD is equal to 0, it means that the two means are equal, and it also means that the two means are not statistically significant, so the invalid vertical line of the SMD forest plot is at 0 on the abscissa scale. Since we did a subgroup analysis, the total values shown at the bottom are what we need to get. In addition, the values within subgroups become less meaningful. Tau-squared, chi-squared, and I^2^ all indicated heterogeneity between studies, and the I^2^ result was more meaningful. I^2^ is used to evaluate the heterogeneity of the results and draw forest diagrams and funnel diagrams (59). It is generally believed that an I^2^ >50% indicates that the research has strong heterogeneity, and vice versa. Degree of freedom (df) refers to the number of variables that can take unlimited values when calculating a statistic. Test for overall effect is reflected by the *p*-value. When *p* is >0.05, the combined data are considered to be not statistically significant (60, 61). Due to the large differences in the demographics, cognitive levels, and serum indicators of the study participants included in the meta-analysis, the foreseeable analysis is heterogeneous. We will also conduct subgroup analysis based on the characteristics of the included studies.

**Table 1:** The Newcastle–Ottawa Scale (NOS) used to evaluate the quality of the included literature, “⋆⋆” means excellent, “⋆” means ordinary, “–” means not mentioned, the total score of the article is not <5 points to be qualified.

**Study**	**Selection**				**Exposure**		**Comparability control for important factor**	**Score**
	**Adequate definition of cases**	**Represen tativeness of cases**	**Selection of controls**	**Definition of controls**	**Ascertainment of exposure**	**Same methods of ascertainment for cases and controls**		
Zhu (35)	⋆	⋆⋆	⋆	⋆	⋆⋆	⋆⋆	⋆	10
Correa (36)	⋆	⋆	⋆	⋆⋆	⋆⋆	⋆	⋆	9
Hoare et al. (37)	⋆	⋆⋆	⋆	–	⋆	⋆	⋆	7
Uban et al. (38)	⋆⋆	⋆	⋆	–	⋆⋆	⋆	⋆	8
Ackermann et al. (39)	⋆	⋆	⋆	⋆	⋆	⋆	⋆	7
Blokhuis et al. (40)	⋆	⋆⋆	⋆	⋆	⋆	⋆	⋆	8
Cysique et al. (41)	⋆	⋆	⋆	⋆	⋆	⋆	⋆	7
Paul et al. (42)	⋆⋆	⋆⋆	⋆	⋆⋆	–	⋆	⋆	9
Underwood et al. (43)	⋆⋆	⋆⋆	⋆	⋆	⋆	⋆	⋆	9
Wakim et al. (44)	⋆	⋆	⋆	⋆	⋆⋆	–	⋆	7
Haynes et al. (45)	⋆	⋆⋆	⋆	⋆	⋆	–	⋆	7
Li et al. (46)	⋆⋆	⋆⋆	⋆	⋆	⋆⋆	⋆	⋆	10
Liang et al. (47)	⋆	⋆	⋆	⋆	⋆	–	⋆	6
Oh et al. (48)	⋆	⋆⋆	⋆	⋆	⋆	⋆	⋆	8
Davis et al. (49)	⋆⋆	⋆⋆	⋆	⋆	⋆	⋆	⋆	9
Watson et al. (50)	⋆⋆	⋆	⋆	⋆	⋆	⋆	⋆	8
Samboju et al. (51)	⋆	⋆	⋆	⋆	⋆	–	⋆	6
Hoare et al. (52)	⋆⋆	⋆⋆	⋆	⋆	⋆	⋆	⋆	8
Kuhn et al. (53)	⋆	⋆	⋆	⋆	⋆	⋆	⋆	7
Ackermann et al. (54)	⋆	⋆⋆	⋆	⋆	⋆	⋆	⋆	8
Zhao et al. (55)	⋆⋆	⋆	–	–	⋆⋆	⋆	⋆	7
Liang et al. (57)	⋆⋆	⋆⋆	⋆	⋆	⋆	⋆	⋆	9

## Results

First, we evaluated the included articles with the NOS, which confirmed to the quality evaluation standards of cross-sectional studies or cohort studies. Each item can be divided into three grades: excellent, ordinary, and not mentioned, corresponding to 2 points, 1 point, and 0 point, respectively. The eligibility standard for literature is 5 points or more. According to statistics, all the 22 documents included in the meta-analysis and system review meet the conditions.

### Demographics

By pooling, these studies included 702 patients with HIV+/HAND and 346 healthy controls. The age range of the patient group was 4–77 years, with an average age of 48.52 years. The age range of the control group was 3–73 years, with an average age of 46.21. Of these studies, three were in adolescents and children, one was in young adults, and the rest were in middle-aged and elderly patients.

### Meta-Analysis

In the CC, patients with HAND and HIV-seropositive have a significant reduction in FA (SMD = −0.57; 95% CI: −1.18 to 0.03), test for overall effect: *Z* = 1.86(*p* = 0.06), with high study heterogeneity (chi-squared = 153.19, df = 9, *p* < 0.00001) and I^2^ = 94% (variation in SMD attributable to heterogeneity). The Tau-squared of between-study variance was 0.12. Among the ten studies included in the analysis, five studies have a confidence intervals of zero.

Also in the CC, patients with HAND and HIV-seropositive have an increase in MD (SMD = −0.04; 95% CI: 0.01 to 0.06), test for overall effect: *Z* = 2.63 (*p* = 0.008), with high study heterogeneity (chi-squared = 76.91, df = 9, *p* < 0.00001) and I^2^ = 88% (variation in SMD attributable to heterogeneity). The Tau-squared of between-study variance was 0.00. Among the 10 studies included in the analysis, six studies have a confidence interval of zero.

Different from the CC, the FX, AC, association fibers, and projection fibers differ greatly in the image acquisition area and ROI selection in various studies. FA and MD values are not suitable for simply taking the arithmetic mean. In statistics, inverse-variance-weighting is a method of performing a weighted average of the measurements of a random variable. Each smaller ROI was weighted by the inverse of its variance in the random effects model of this study. This method minimizes the variance of the mean and makes the results more accurate. For studies with only the median, regardless of whether the samples meet the normal distribution or not (62), first perform Box-Cox transformation and then use an online calculator to estimate the sample standard deviation and sample mean for the transformed study. The estimator results are more accurate (63). Among other WMF, patients with HAND and HIV-seropositive have a reduction in FA (SMD = −1.18; 95% CI: −1.82 to −0.55), test for overall effect: *Z* = 3.67 (*p* = 0.0002), with high study heterogeneity (chi-squared = 186.93, df = 10, *p* < 0.00001) and I^2^ = 95% (variation in SMD attributable to heterogeneity). The Tau-squared of between-study variance WMF was 1.05. Among the 11 studies included in the analysis, six studies have a confidence interval of zero.

**Figure 2:**
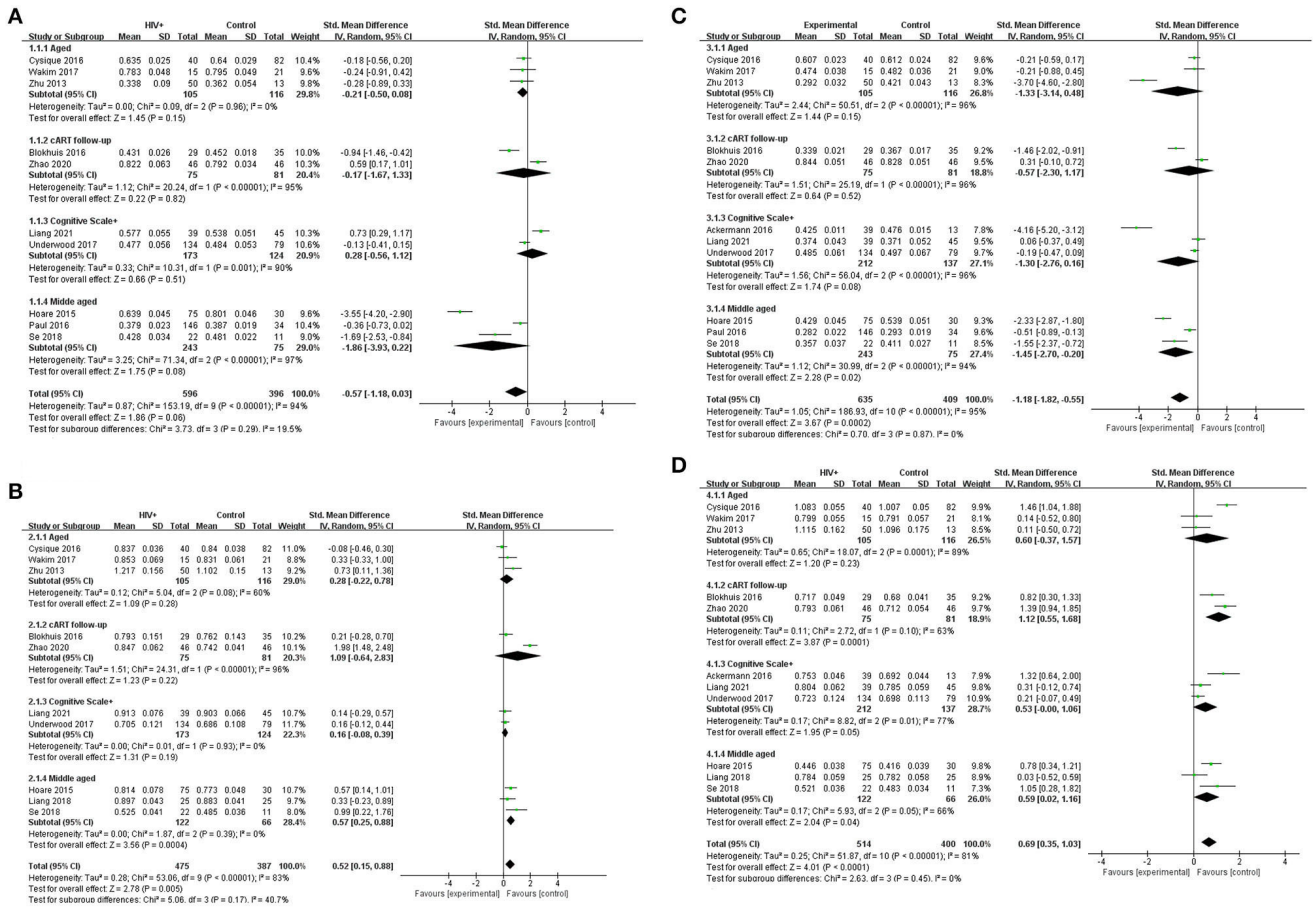
Meta-analysis results of FA and MD between the HIV-positive group and the control group. **(A)** FA in CC. **(B)** MD in CC (*10^3^). **(C)** FA among other WMF. **(D)** MD among other WMF (*10^3^).

Among others, patients with HAND and HIV- seropositive have a notable increase in MD, and patients with HAND and HIV-seropositive have a significant reduction in FA (SMD = 0.69; 95% CI: 0.35 to 1.03), test for overall effect: *Z* = 4.01 (*p* < 0.0001), with high study heterogeneity (chi-squared = 153.19, df = 9, *p* < 0.00001) and I^2^ = 81% (variation in SMD attributable to heterogeneity). The Tau-squared of between-study variance was 0.25. Among the 10 studies included in the analysis, five studies have a confidence interval of zero (Figure 2).

**Figure 3:**
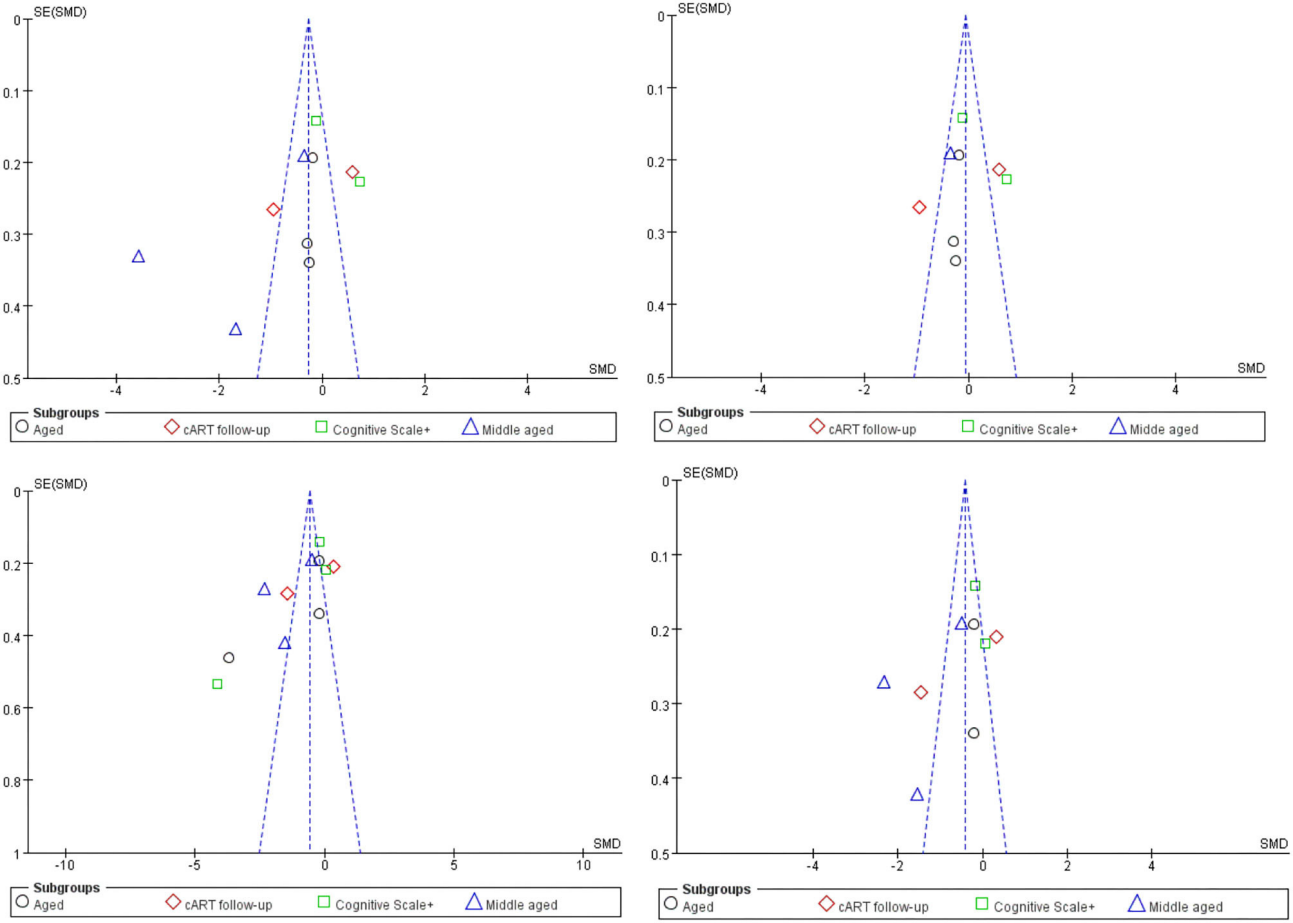
Funnel plot of meta-analysis results. These four figures represent FA in CC, FA in CC except (37, 56), FA among other WMF, FA among other WMF [except (35, 39)].

**Figure 4:**
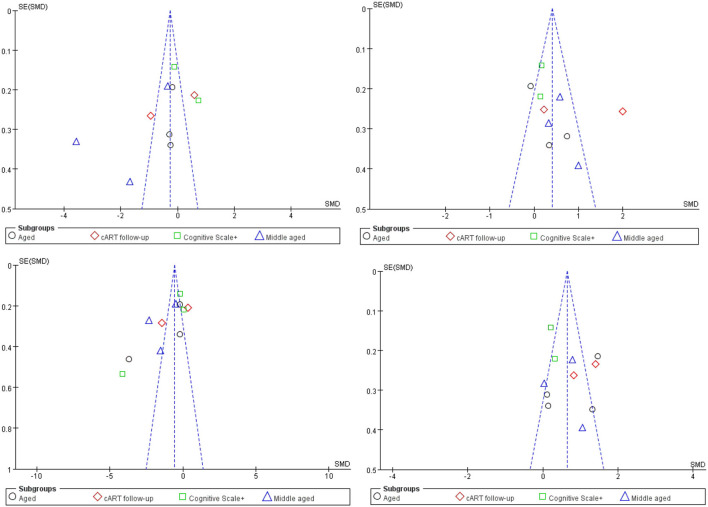
Funnel plot of meta-analysis results. These four figures represent FA in CC, MD in CC, FA among other WMF, MD among other WMF.

Obviously, whether in the CC or WMF, the FA value of patients with HAND decreased whereas the MD increased, and the CC changed to a greater degree relative to other WMF. However, the included analysis research has a large heterogeneity, which has a great impact on the reliability of the results. The main source of the aforementioned heterogeneity is the change of acquisition parameters or biological variables, including field strength, b value, number of diffusion directions, and application of TBSS, etc. We divided the studies included in the analysis into four subgroups according to the age range of the study subjects, the duration of cART, and the cognitive scale. Based on the statistical information in Table 2, we grouped three studies (35, 41, 44) into older subgroups because their participants were older in several studies. We grouped the two studies (40, 55) into the same subgroup because they were the only follow-up studies in the study. Since these three studies (39, 43, 57) were fully tested on the NP and cognitive scales, we assigned them to the same subgroup. The last few studies (37, 42, 47, 56) differed considerably, but they had in common the relatively young age of the investigators, which led them to be grouped into the same subgroup. It is worth noting that our groupings are based on the greatest commonality between studies, and there may be large differences between studies within the group on other factors that are still outside the basis of grouping. After subgroup analysis, the analysis results of the four groups are as follows: FA in CC, test for subgroup differences: I^2^ = 19.5(chi-squared = 3.73, df = 3, *p* = 0.29). MD in CC, test for subgroup differences: I^2^ = 40.7 (chi-squared = 5.06, df = 3, *p* = 0.17). FA among others WMF test for subgroup differences: I^2^ = 0 (chi-squared = 0.70, df = 3, *p* = 0.299). MD among other WMF, test for subgroup differences: I^2^ = 0 (chi-squared = 2.63, df = 3, *p* = 0.45). The significant reduction in the heterogeneity of the subgroup analysis indicates the correctness of the subgroups. It also proves that the above grouping basis is the source of heterogeneity. The funnel plot showed that the asymmetry between studies was more pronounced in FA values than in MD values, because the two funnel plots associated with MD values have more studies with built-in confidence intervals than the two funnel plots associated with FA values, suggesting a greater publication bias. We used the cut-and-complement method to eliminate the studies with large deviations from the mean. We tried to exclude studies that were far from the confidence interval in the two analyses related to the FA value [FA in CC except (37, 56), FA among other WMF, except (35, 39)] and obtained the results of the funnel plot (Figure 3). The results showed that the combined effect size estimates did not change significantly, and the funnel plot did not change significantly. This indicates that there is publication bias in the FA value among studies, but it is not obvious, the publication bias has less effect, and the results are relatively robust (Figure 4).

**Table 2:** Statistical table of studies included in the meta-analysis.

**References**	**Type of study**	**Participant, age**	**Age**	**Field strength**	**TE**	**TR**	***b*** **-value**	**Analysis**	**NP/cognitive scale**	**Treatment**	**ROI**
Oh et al. (56)	Cross-sectional	22HIV+, 11Cotrol	54.5 ± 2.5	3T	109	10,000	3,000.7	TBSS	+	Unknown	CC,CST,SLF,IFOF,ATR
Zhao et al. (55)	Follow-up	46HIV+, 46Cotrol	43.96 ± 13.21	1.5T	97	5,000	1,000		+	cART	Whole brain
Wakim et al. (44)	Cross-sectional	15HIV+, 21Cotrol	50.43 ± 5.42	3T	56	7,600	800	MNI	+	HAART	ATR,SLF,CC,ILF,UF,IFOF
Ackermann et al. (39)	Cross-sectional	39HIV+, 13Cotrol	5.56 ± 0.41	3T	86	9,500	1,000		+	ART	ILF,SLF,IFOF,UF,CST
Underwood et al. (43)	Cohort study	134HIV+, 79Cotrol	56.2 ± 3.25	3T				TBSS	+	ART	Whole brain
Liang et al. (57)	Cross-sectional	39HIV+, 45Cotrol	47.15 ± 12.01	3T	88	3,700	1,000		+	cART	Whole brain
Liang et al. (47)	Cross-sectional	25HIV+, 25Cotrol	45.2 ± 2.3	3T	88	3,700	1,000	LDDMM	–	cART	CC,ACR,SCR,PCR,ALIC,SFO
Zhu (35)	Cross-sectional	50HIV+, 13Cotrol	50.5 ± 8.5	1.5T	75	7,000	1,000.2	TBSS	+	ART	CC,ACR,SCR,PCR,ALICILF,SLF,CST
Paul et al. (42)	Cross-sectional	146HIV+, 34Cotrol	28.5 ± 4.9	3T	103	10,000	1,000	DTI-TK	–	cART	CC,CING,ATR,UNC
Blokhuis et al. (40)	Follow-up	29HIV+, 35Cotrol	12.6 ± 3.5	3T					–	cART	CC,GCL,OPL
Cysique et al. (41)	Cross-sectional	82HIV+, 40Cotrol	54.8 ± 6.6	3T			1,000	TBSS	+	Unknown	CC,SLF,ALIC,FX,CST
Hoare et al. (37)	Cross-sectional	75HIV+, 30Cotrol	9.9 ± 2.4	3T	88	8,800	1,000	TBSS	–	ART	CC,ACR,SCR,PLIC,EC

*The unit of age is year. The units of TR and TE are ms. The unit of b value is mm/s^2^*.

Although the main reason for the high heterogeneity was not found in the funnel chart, we still summarized the differences in acquisition parameters, demographics, serology, and treatment time between studies in the extraction stage before the included studies. The field strength (range 1.5T−3T), number of diffusion directions (range 6–64), age of participants (range 4–69 years), nadir CD4 count (range 33.5–221.8^*^10^6^/L), current CD4 count (range 230.4–980^*^10^6^/L), cART duration (range 0–72 months), cognitive scale test (Wisconsin Cognitive Test, Global Defect Score, and Frascati Manual). There is high heterogeneity in FA and MD values among the four groups of studies. These differences in white matter microstructures between groups may be caused by changes in the above-mentioned image acquisition parameters or biological variables.

**Figure 5:**
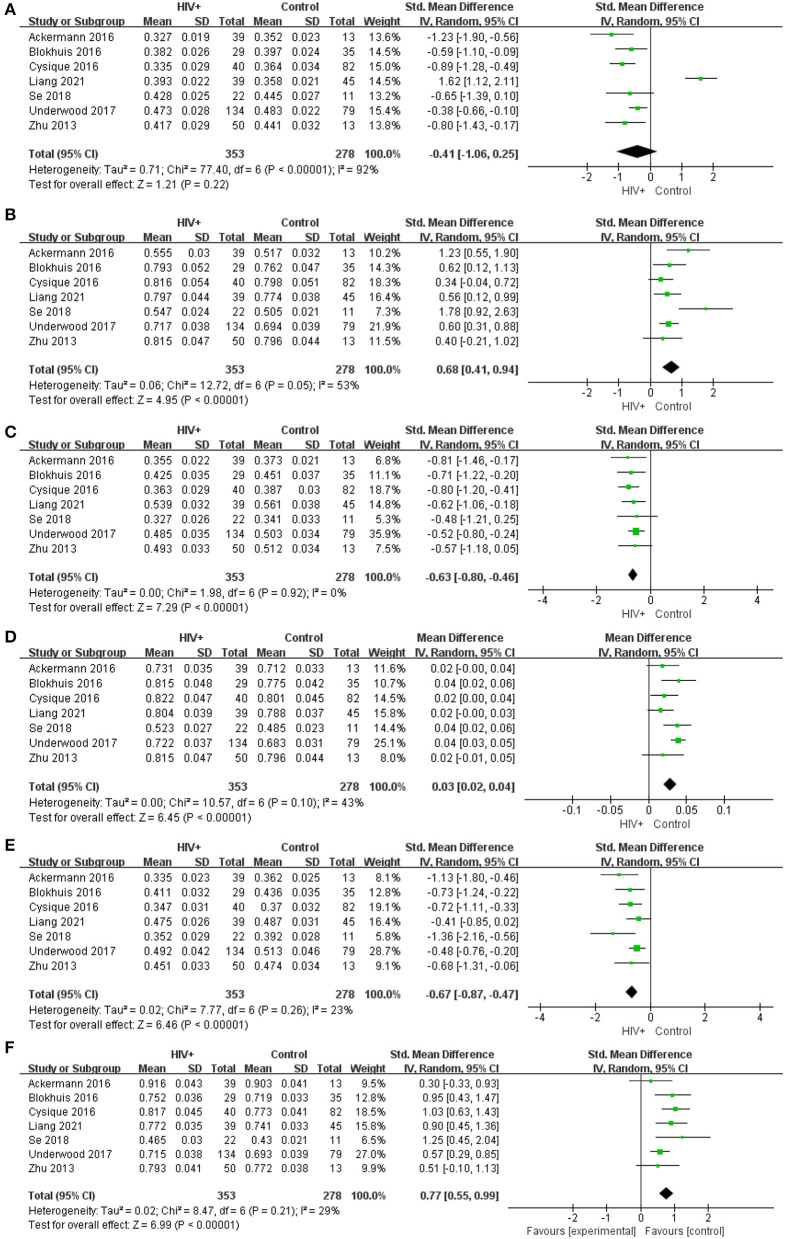
Meta-analysis results of FA and MD between the HIV positive group and the control group. **(A–F)** Represent FA in CST, MD in CST (*10^−3^), FA in SLF, MD in SLF (*10^−3^), FA in IFOF, MD in IFOF (*10^−3^).

Meta-analysis results of FA and MD values in CST, SLF, and IFOF show (Figure 5): FA value in CST (SMD = −0.41; 95% CI: −1.06 to 0.25), test for overall effect: *Z* = 1.21(*p* = 0.22), with high study heterogeneity (chi-squared = 77.40, df = 6, *p* < 0.00001)and I^2^ = 92%; MD value in CST (SMD = 0.77; 95% CI: 0.55 to 0.99), test for overall effect: *Z* = 6.99 (*p* < 0.00001), with low study heterogeneity (chi-squared = 8.47, df = 6, *p* = 0.21) and I^2^ = 29%; FA value in SLF (SMD = −0.67; 95% CI: −0.87 to −0.47), test for overall effect: *Z* = 6.46 (*p* < 0.00001), with low study heterogeneity (chi-squared =7.77, df = 6, *p* = 0.26) and I^2^ = 23%; MD value in SLF (SMD = 0.03; 95% CI: 0.02 to 0.04), test for overall effect: *Z* = 6.45 (*p* < 0.00001), with low study heterogeneity (chi-squared =10.57, df = 6, *p* = 0.10) and I^2^ = 43%; FA value in IFOF (SMD = −0.63; 95% CI: −0.80 to −0.46), test for overall effect: *Z* = 7.29(*p* < 0.00001), with low study heterogeneity (chi-squared =1.98, df = 6, *p* = 0.92) and I^2^ = 0; MD value in IFOF (SMD = 0.68; 95% CI: 0.41 to 0.94), test for overall effect: *Z* = 4.95 (*p* < 0.00001), with low study heterogeneity (chi-squared =12.76, df = 6, *p* = 0.05) and I^2^ = 53%. The results revealed that the heterogeneity of FA values and MD values in the rest regions of interest was low, except for the higher heterogeneity of FA values in the CST region and the slightly higher MD values in the IFOF region. This suggests that although there are differences between studies in larger regions such as the CC or other projection fibers, associative fibers, the consistency of findings between the selected smaller regions of interest is high.

Despite the studies included in the analysis have carried out tracing of WMF in the whole brain area, the differences in measurement parameters such as b value and diffusion directions have led to the white matter in different areas under the premise of similar biological conditions. Fiber DTI parameters still have high heterogeneity, which is unavoidable in subgroup analysis. In terms of the trend of this difference, the integrity of the CC is better than other commissural fibers, association fibers, and projection fibers. Therefore, except for the CC, our unified analysis of other WMF will minimize the differences between different studies due to different image acquisition parameters and then infer the main source of heterogeneity between studies. There is less heterogeneity between studies in smaller ROIs than in CCs or other larger white matter tracts, which may suggest that differences in subjects, parameters, and methods between studies have less effect on smaller ROIs, whereas such differences in CC would have additive effects, increasing heterogeneity between studies. Taking into account some regional differences in the measurement, the observed heterogeneity of the CC and other WMF between FA value and MD value groups may also be due to the sampling of different WMFs in different studies. To more closely study the changes in the CC of FA value and MD value that are related to the cognitive impairment status under HIV infection, we repeated the measurement several times and adopted the fixed-effects model and the random-effects model, respectively, and obtained higher levels in CC, FA value, and lower MD value. As far as the inside of the CC is concerned, the degree of change in the genu and body is higher than that of the splenium. In addition to the differences in DTI parameters between studies, neurocognitive function scales and age are decisive factors for high heterogeneity between studies, and factors such as cART drug selection and duration, and the number of CD4+ T cells also play an important role.

### System Review

Among the 10 articles excluded from the meta-analysis, eight studies suggested that the difference between FA value and or MD value between patients with HAND and the control group was statistically significant, and one study concluded that AD value at the ANI stage the reduction was limited to the left anterior thalamus the fronto-occipital lobe, and the left SLF, and the FA value, MD value, RD value, and AD value were not significant (36, 49, 51, 64). A total of four studies used the Wisconsin Card Sorting Test. In the test, the AD value was negatively correlated with the language fluency test and positively correlated with the attention/work area. The MD value of the CC and the memory (learning/delayed recall) score and information processing speed scores are negatively correlated. Fine motor scores are negatively correlated with the MD and AD values of the CC (52). DTI studies on the changes in the microstructure of white matter in patients with HIV found that in early HAND, the damaged part is the CC. As the disease progresses: extensive damage to the cerebral white matter, mainly in the CC, fronto-parietal lobe, corona radiata, center of semiovale, the so-called periventricular white matter area. Li's research believes that DTI parameters effectively quantify the changes in the CC and the radiation coronal area (the MD value is significantly increased). In patients with ANI, WM has obvious damage, and the WMFs around the ventricle are often the first area. The pathological basis of this injury is axon damage rather than demyelinating disease (52). Graph theory-based studies of WM topology revealed that the untreated group had lower aggregation coefficients, weaker structural segregation, integration, and connectivity strengths. The overall clustering coefficient was lower in the treatment group, indicating a segregated brain network and lower nodal degree in the left thalamus.

A total of three studies involved follow-up and longitudinal studies. The participants included children, adolescents, and the elderly (45, 46). In general, white matter microstructure damage has been found in people of all ages. After the same period of cART, the differences in HIV serum status, viral load, and cognitive function between different age groups are statistically significant. In elderly patients with HAND, it is reasonable to conclude that individuals accelerate aging after excluding other neurodegenerative diseases and cerebrovascular diseases. The changes in DTI parameters are manifested as a significant decrease in FA and a significant increase in MD of the CC and internal capsules (ICs). For the children group, the results of the directional neurodevelopmental test underestimated the degree of damage to the white matter fiber bundles reflected by the changes in DTI parameters. The decrease in FA value and the increase in MD value were most obvious in IFOF (65). Kuhn's study found that there is a significant interaction between the increase in age and the damage of HIV to WMF, which is manifested in the older patient group and the younger patient group having significantly lower FA values and higher MD values, AD values, and RD values. Compared with the elderly healthy control group, the elderly patient group also has a similar trend, but the changes in the parameters are more minor (45).

There are two studies that quantitatively analyze the length and integrity of WMF. Woodruff measured the whole brain fiber length and the length of 6 white matter tracts on 135 patients with HIV-positive and 21 healthy controls and found that the whole brain fiber length was significantly reduced, but the average of the 6 white matter tract lengths was not significantly different (54). Although the patients were treated with cART, the length of whole brain fibers was significantly reduced in patients with HIV-positive (including some patients with ANI) and healthy controls, especially in anterior thalamic radiation (ATR), inferior longitudinal fasciculus (ILF), lower review, and bundle under the forehead. However, it is worth noting that there is no correlation between the shortening of fiber length and poor cognitive function and serum viral indicators. The white matter integrity of the HAND group was significantly poorer, especially in the PTR, IC, EC, and CC (66).

All study participants are on antiretroviral therapy. Some studies have found that as the duration of antiretroviral therapy increases, the FA value will increase and the MD value will decrease compared to the acute infection period of HIV (49, 64). However, as the duration of continuous treatment continues to extend, FA values will decrease and MD will increase, accompanied by impairment of cognitive function, and this trend will become more obvious as the duration of treatment is further extended. The range of changes in DTI parameter values in the brain will gradually expand (49).

Emotional change is often one of the manifestations of cognitive impairment, and it is also a symptom that is relatively easy to find clinically. Some studies have taken a different approach (64). By conducting neurocognitive scale and anxiety level tests, patients with HAND have significant differences in the four cognitive function areas compared with HIV-negative controls. The incidence of anxiety in patients with HAND is significantly higher. In the control group, the degree of anxiety is correlated with the change of DTI parameters. Poor cognitive ability is correlated with higher MD value, lower FA value, and more serious anxiety state (64).

## Discussion

Meta-analysis results showed that compared with the control group, the DTI parameters of the whole brain of patients with HAND showed a trend of decreasing FA value and increasing MD value. The change value of the CC is more obvious than that of other fibers, which is also a strong evidence of the microstructure damage of white paper fibers (66–68). However, there is a large heterogeneity among the studies included in the analysis, and the difference in sequence parameters and brain region selection between different studies is not sufficient to explain this heterogeneity. When we conducted a subgroup analysis based on the differences in the demographics, cART duration, and neurocognitive scales of the study subjects, the heterogeneity of the study decreased significantly, and no significant publication bias was found. This proves that the grouping basis of the above subgroups is the source of heterogeneity in the analysis. The DTI parameter metrics heterogeneity of the CC is still higher than that of other WMF after subgroup analysis, suggesting that the source of the CC heterogeneity may be more complicated, which is worthy of further consideration and exploration (69–71). Although high heterogeneity still exists, there is still a significant correlation between changes in white matter fiber structure and changes in DTI parameters, and changes in white matter fiber structure are one of the necessary conditions for the diagnosis of HAND. Although the research design, research objects, and statistical methods included in the analysis are not the same, the correlation between the above three has been verified in all studies.

A large number of studies have proved that the changes in DTI parameters of neurodegenerative diseases including HAND are mainly FA and MD values (72). The changes in AD and RD values vary greatly between studies, so they were not included in the meta-analysis. However, the current research generally believes that there is a negative correlation between the change of RD value and the FA value, and that there is a positive correlation between the change of AD value and the change of MD value (73, 74). In the pathological basis of neurodegenerative diseases, the change of RD value is related to myelin damage, and the change of AD value is related to neuronal axon damage (75). The change of RD value is often earlier than the AD value, suggesting that damage of axon is very likely earlier than myelin damage. However, it is very difficult for traditional imaging methods to find damage of axon, which means that patients with HAND have neuronal damage or even death before they have positive imaging findings (76, 77). However, it is worth mentioning that the change of FA value and MD is prompting the change of white fiber structure, which also illustrates the advantages of DTI. However, the diagnosis of HAND requires a certain degree of structural change and functional changes (78). We cannot make diagnosis based on the changes of DTI parameters. Therefore, in addition to DTI, there are more neuropsychological examinations and imaging examinations to support this diagnosis.

The changes in DTI parameter values of patients with HAND in each stage are different. Cysique found that the DTI parameter value of the white matter fiber tracts of patients in the ANI stage was close to normal, and only the difference in the MD value of anterior limb of internal capsules (ALICs) was statistically significant (79). Compared with healthy controls and patients with ANI, patients in MND and HAD had lower FA values of the cingulate cortex and FX, and higher MD values of the FX and EC. However, patients with stronger CNS penetrating antiretroviral drugs have higher CD4+ T cells, lower MD values, and higher FA values (41, 80, 81). Oh (56) believes that compared with the healthy group, the decline in information processing speed, memory, executive ability, and fine motor function of patients with HAND in the ANI and MND stages is significantly related to the destruction of the integrity of the white matter fiber microstructure of the frontal and parietal lobe. Compared with patients with HIV-positive without cognitive impairment, patients with HAND have significant changes in the structure of the bilateral SLF, which is also related to cognitive decline.

Unlike common cognition, neurocognitive impairment caused by HIV infection can also occur in adolescents and children. Hoare found that among children aged 6–16, whether they experience cART or not, the integrity of WMF and neurocognitive functions is impaired, and a considerable number of people suffer from HIV-encephalopathy (HIVE). Compared with the uninfected group, patients in the infected group had higher MD values of bilateral EC, left FX, CC, and left former corona radiata area (48). Compared with patients who received cART treatment, patients who received treatment had higher MD values of the left EC and CC (37). Compared with acquired patients with HIV, patients with HIV in the perinatal period have a higher risk of changes in white matter structure (82, 83). Uban's study (84) concluded that, in addition to the AD value, the differences in FA, MD, and RD values between perinatal patients with HIV and healthy controls are statistically significant. The viral load is significantly correlated with the change of the DTI parameter value of the IFOF on the right.

For the elderly, the source of cognitive impairment in patients with HIV-positive not only comes from inflammation in the brain caused by viral infection, but may also come from other neurodegenerative diseases. This may be a huge blow to the cognitive function of the elderly (38, 85). Underwood (86) compared the cognitive function test results of 134 patients with HIV-positive and 79 healthy controls and found that the positive group had impairments in 4 cognitive domains (6 domains in total). The white matter volume of the positive group did not decrease significantly, but the FA value decreased, and the MD value and RD value increased, indicating that the white matter microstructure was damaged. Watson's study (43) showed that with the prolonged antiretroviral therapy, there was no difference in white matter intensity between the HIV-infected group and the healthy control group. However, for elderly individuals, the burden of WMH is associated with higher cardiovascular risks and worse DTI parameter values are correlated, and the latter is correlated with worse neurocognitive function test performance.

Partial immune reconstitution is an inevitable process in patients with HIV undergoing antiretroviral therapy. However, some studies have suggested that partial immune reconstitution also seems to be one of the conditions that induce cognitive impairment. Zhu's research (50) suggests that the range of changes in DTI parameters increases with the prolonged infection time, and the decrease in FA is mainly in the GCC, bilateral corona radiata area, and bilateral cingulate cortex, but the changes in FA values are only more pronounced in the MND stage (35). Compared with the ANI stage, the MD value of patients in the MND stage extends from the bilateral rear frontal lobes to the bilateral prefrontal lobes. The SLF, ILF, and the subfrontal occipital fascicle are important components of the language pathway, and the CC is another language control area. The changes in the DTI parameter values in the above-mentioned areas are the manifestations of language function impairment (87, 88).

The neurocognitive scale is an important tool for evaluating cognitive impairment, and it cooperates with DTI to complete the diagnosis of HAND. Dreyer used 20 different methods to diagnose HAND on 148 HIV-positive participants and obtained patient's white matter and gray matter images. The accuracy of the 20 different methods was between 20 and 97%, and a single neurocognitive amount table assessment or DTI examination and the accuracy of reflecting the cognitive deficits of patients with HIV are relatively poor (89). The currently commonly used neurocognitive scales include the Global Defect Score (GDS), Wisconsin Cognitive Test (WCT), Frascati Standard, and Montreal Cognitive Assessment (MoCA) (90, 91). Mukherjee (92) considers that the accuracy and credibility of MoCA's diagnostic evaluation for different degrees of HAND is higher than the traditional Frascati standard after the correction of demographics, but there are still shortcomings such as complex statistical methods and inconvenience for large-scale development.

Current researches believed that retinopathy is related to brain white matter fiber damage, so some research has taken a different approach (93, 94). Blokhuis (95) studied the differences in the microstructure of the retina and WMF between patients with HIV and the control group and found that the lower thickness of the fovea and surrounding retina in patients with HIV-positive was associated with lower FA values and higher MD and RD values. The thickness of the retina is related to the damage of white matter and has nothing to do with the decrease of brain volume, suggesting that the two have a common pathogenesis. HIV may jointly affect the maturation of the optic nerve and white matter (40).

Smoking or the abuse of other addictive drugs (such as marijuana, cocaine, methamphetamine, etc.) may aggravate the cognitive impairment of patients with HAND, and more and more studies are now focusing on this (96, 97). Liang (98) compared smoking patients with HIV-positive with non-smoking patients with HIV-positive and found that smoking patients with HIV-positive have higher diffusivity in GCC, SCC, and anterior corona radiata area, which indicates a decline in cognitive function. The higher AD value predicts that smoking patients with HIV-positive have slower information processing speed and lower attention, indicating that smoking is a risk factor for HAND. Wakim's study (47) concluded that patients with HIV-positive with cocaine dependence have a higher risk of mortality and cognitive impairment, which may be related to increased myelin damage. Cordero (44) believes that HIV is related to the white matter integrity of the entire brain, but cocaine dependence does not seem to exacerbate the effects of HIV. In the future, there will be more relevant research attention to further explore the correlation between other cognitive impairment risks and HAND.

## Limitation

Diffusion tensor imaging is the most classic and clinically widely used diffusion imaging technique. It has good sensitivity for white matter microstructural abnormalities, but its specificity is not high enough. In DTI data interpretation, the most common misconceptions relate to scalar (such as AD, FA, MD, and RD, and so on) results. In general, higher MD values and lower FA values indicate compromised fiber integrity due to increased diffusion in the main fiber direction and loss of fiber myelination (99). However, sometimes, the results are inaccurate, and the relationship between abnormal levels and structural changes is not necessarily related, so it is necessary to combine other test results and supplementary data. Compared with the traditional DTI and DKI models, the neurite direction dispersion and density imaging (NODDI) (100) designed in the recent years can more directly and accurately reflect the microstructure of brain tissue. For our meta-analysis, although the number of study samples met the criteria, it was not sufficient. This may increase the risk of publication bias between studies and make our results appear less robust (101). We will further collect the latest articles in the future, which is also a validation of our analysis results.

## Conclusion

Through meta-analysis and system review, we have obtained the changes in DTI parameter metrics of patients with HAND. Although there is still great heterogeneity between the studies, the changes occurred in parameter values (decrease in FA value and increase in MD value) and cognition. There is a significant correlation with functional impairment, which is more obvious in the CC. The degree of cognitive impairment is also related to the factors such as cART duration, neurocognitive scale selection, serum viral load, and CD4+T cell level. DTI can be an important tool in the diagnosis of HAND, but it has disadvantages such as poor specificity, many artifacts, and susceptibility to cross-fiber interference. Moreover, HAND cannot be accurately diagnosed by DTI examination or neurocognitive scale test alone. It must be combined with other imaging methods and clinical laboratory tests to effectively evaluate cognitive impairment and structural changes in the brain in patients with HIV.

## Data Availability Statement

The original contributions presented in the study are included in the article/supplementary material, further inquiries can be directed to the corresponding author/s.

## Author Contributions

JM is responsible for article writing and data collection. FX is responsible for finding references. XY is responsible for the use of meta-analysis methods. HL is responsible for guiding the ideas and writing directions of the article. All authors contributed to the article and approved the submitted version.

## Funding

This work was supported by the National Natural Science Foundation of China (Grant Nos. 81771806 and 61936013), the Beijing Natural Science Foundation (7212051), AI key R&D project of Beijing Municipal Science and Technology Commission (Z211100003521003), Ministry of Science and Technology International Cooperation Project (2020ZX100), and Key R&D chief project of the Ministry of Science and Technology (2020ZX178).

## Conflict of Interest

The authors declare that the research was conducted in the absence of any commercial or financial relationships that could be construed as a potential conflict of interest.

## Publisher's Note

All claims expressed in this article are solely those of the authors and do not necessarily represent those of their affiliated organizations, or those of the publisher, the editors and the reviewers. Any product that may be evaluated in this article, or claim that may be made by its manufacturer, is not guaranteed or endorsed by the publisher.

## Abbreviations

DTI: diffusion tensor imaging
HAND: HIV-associated neurocognitive disorder
AIDS: acquired immunodeficiency syndrome
cART: combined antiretroviral therapy
ANI: asymptomatic neurocognitive impairment
MND: mild neurocognitive disorder
HAD: HIV-associated dementia
FA: fractional anisotropy
MD: mean diffusivity
AD: axial diffusion
RD: radical diffusion
CNS: central nervous system
SMD: standardized mean difference
CC: corpus callosum
WMF: white matter fibers
GCC: genu of the corpus callosum
SCC: splenium of corpus callosum
SLF: superior longitudinal fasciculus
ILF: inferior longitudinal fasciculus
IFOF: inferior fronto-occipital fasciculus
CST: corticospinal tract
UF: bilateral uncinate fasciculus
EC: external capsules
IC: internal capsules
FX: fornix
PTR: posterior thalamic radiation
ATR: anterior thalamic radiation
AC: anterior commissure
NP: neuropsychology
ROI: region of interest
VBA: voxel-based analysis
TBSS: tract-based spatial statistics
FBA: Fixel-based analysis
NOS: Newcastle–Ottawa Scale
HIVE: HIV-encephalopathy
GDS: Global Defect Score
WCT: Wisconsin Cognitive Test
MoCA: Montreal Cognitive Assessment
NODDI: neurite direction dispersion and density imaging.
